# Supplementary material for: Airway Care Interventions for Invasively Ventilated Critically Ill Adults—A Dutch National Survey
Source: J Clin Med. 2021 Jul 30;10(15):3381. doi: 10.3390/jcm10153381 (PMC8347919; doi:10.3390/jcm10153381)
Supplement: Supplementary file 1 [file jcm-10-03381-s001.zip › jcm-1255834-supplementary.pdf]

eSupplement

eMethods

Proportions and differences with 95% confidence interval of airway care interventions between type of hospitals or size of ICU were reported. To test the association of hospital type (academic/teaching compared to general hospitals) or ICU-size (> 20 beds compared to < 20 beds) with the use of heated humidification, nebulization, manual hyperinflation and mechanical insufflation-exsufflation separate logistic regression models with hospital type and ICU-size as predictors in models with the interventions as outcomes: heated humidification (on indication (1) = viscous mucus, routine(0) = always and ventilation > 24 hrs); nebulization (routine = 0 and on indication = 1); manual hyperinflation (use of MH (routine and on indication) = 1, never = 0), mechanical insufflation-exsufflation (use of MI-E (routine and on indication) = 1, never = 0). We fitted the models without an intercept to present all coefficients directly and to test the hypothesis that each individual coefficient is zero and not the pairwise comparison between coefficients. We report odds ratio’s and their 95% confidence intervals).

eResults

Supplementary Table S1. Proportions and their differences.

	**beds ≤ 20**	**beds > 20**	**difference**
Heated humidification	0.15 (0.06 to 0.29)	0.15 (0.04 to 0.35)	0 (-0.2 to 0.16)
Nebulization	0.61 (0.45 to 0.75)	0.5 (0.3 to 0.7)	0.11 (-0.13 to 0.34)
MH	0.72 (0.57 to 0.84)	0.96 (0.8 to 1)	-0.24 (-0.4 to -0.07)
MI-E	0.24 (0.13 to 0.39)	0.19 (0.07 to 0.39)	0.05 (-0.17 to 0.23)
	**Academic-teaching**	**General**	**difference**
Heated humidification	0.18 (0.08 to 0.34)	0.12 (0.03 to 0.27)	0.07 (-0.11 to 0.24)
Nebulization	0.63 (0.46 to 0.78)	0.5 (0.32 to 0.68)	0.13 (-0.1 to 0.35)
MH	0.87 (0.72 to 0.96)	0.74 (0.56 to 0.87)	0.13 (-0.05 to 0.32)
MI-E	0.32 (0.18 to 0.49)	0.12 (0.03 to 0.27)	0.2 (0 to 0.38)

Supplementary Table S2. Basics of the proportions.

**Intervention**	**beds ≤ 20**	**beds > 20**
Heated humidification	7/46	4/26
Nebulization	28/46	13/26
MH	33/46	25/26
MI-E	11/46	5/26
	**Academic-teaching**	**General**
Heated humidification	7/38	4/34
Nebulization	24/38	17/34
MH	33/38	25/34
MI-E	12/38	4/34

Supplementary Table S3

**Models and predictors**	**OR (95% CI)**
**Heated humidification (routine = 0; on indication = 1)**	
- Academic-teaching hospital	0.23 (95% CI 0.09 to 0.48) *
- General hospital	0.13 (95% CI 0.04 to 0.34) *
> 20 ICU-beds	1.01 (95% CI 0.24 to 3.75)
**Nebulization (routine = 0; on indication = 1)**	
- Academic-teaching hospital	1.71 (95% CI 0.90 to 3.40)
- General hospital	1 (95% CI 0.51 to 1.97)
> 20 ICU-beds	0.64 (95% CI 0.24 to 1.7)
**Manual hyperinflation (never used= 0; use of MH = 1)**	
- Academic/teaching hospital	6.6 (95% CI 2.82 to 19.28) *
- General hospital	2.78 (95% CI 1.34 to 6.29) *
> 20 ICU-beds	9.85 (95% CI 1.78 to 184.82) *
**Mechanical in-exsufflation MI-E (never used= 0; use of MI-E= 1,)**	
- Academic/teaching hospital	0.46 (95% CI 0.22 to 0.89) *
- General hospital	0.13 (95% CI 0.04 to 0.34) *
> 20 ICU-beds	0.76 (95% CI 0.21 to 2.4)
* Significant (if the lower and upper value of the 95% confidence interval are both below or above the 1 this indicates a significant OR)	

Heated humidification

Both types of hospitals, academic/teaching and general hospitals were associated with routine use of heated humidification. The size of the ICU was not associated with its use.

Nebulization therapy

There were no associations of hospital type or ICU-size regarding the use of nebulization therapy.

Manual hyperinflation

Both general and academic/teaching hospitals answers showed a positive association with manual hyperinflation use. In addition, larger ICUs were associated with more manual hyperinflation use.

MI-E

Both hospital types were associated with low use of MI-E. There was no association with size of ICU regarding MI-E.
